# Construction of a mApple-D6A3-mediated biosensor for detection of heavy metal ions

**DOI:** 10.1186/s13568-020-01154-9

**Published:** 2020-12-07

**Authors:** Yangyang Ji, Feifei Guan, Xin Zhou, Xiaoqing Liu, Ningfeng Wu, Daling Liu, Jian Tian

**Affiliations:** 1 School of Life Science and Technology, Jinan University, Guangzhou, 10559 Guangdong China; 2 Biotechnology Research Institute, Chinese Academy of Agricultural Sciences, Beijing, 100081 China

**Keywords:** Biosensor, mApple-D6A3, Heavy metal, Detection

## Abstract

Pollution of heavy metals in agricultural environments is a growing problem to the health of the world’s human population. Green, low-cost, and efficient detection methods can help control such pollution. In this study, a protein biosensor, mApple-D6A3, was built from rice-derived Cd^2+^-binding protein D6A3 fused with the red fluorescent protein mApple at the N-terminus to detect the contents of heavy metals. Fluorescence intensity of mApple fused with D6A3 indicated the biosensor’s sensitivity to metal ions and its intensity was more stable under alkaline conditions. mApple-D6A3 was most sensitive to Cu^2+^, then Ni^2+^, then Cd^2+^. Isothermal titration calorimetry experiments demonstrated that mApple-D6A3 successfully bound to each of these three metal ions, and its ability to bind the ions was, from strongest to weakest, Cu^2+^  > Cd^2+^  > Ni^2+^. There were strong linear relationships between the fluorescence intensity of mApple-D6A3 and concentrations of Cd^2+^ (0–100 μM), Cu^2+^ (0–60 μM) and Ni^2+^ (0–120 μM), and their respective R^2^ values were 0.994, 0.973 and 0.973. When mApple-D6A3 was applied to detect concentrations of heavy metal ions in water (0–0.1 mM) or culture medium (0–1 mM), its accuracy for detection attained more than 80%. This study demonstrates the potential of this biosensor as a tool for detection of heavy metal ions.

## Key points

1. A protein biosensor for detecting heavy metals was constructed.
2. Linear correlation was observed between fluorescence intensity and ion concentration.
3. The accuracy for detection heavy metals in liquid samples attained more than 80%.

## Introduction

Pollution by heavy metals, such as Cd^2+^, Hg^2+^, Pb^2+^, As^2+^, Cr^2+^, Ni^2+^ and Cu^2+^, in some agricultural soils in the world is a serious issue (Duan and Tan 2013; Li et al. 2014, 2020). Crops, especially rice, grown in these soils are of particular concern because they can cause great harm to the health of the animals, including humans, that consume the products from these crops (Brewer 2015; Zhai and Shang 2007). When excessively accumulated in the body, heavy metals will irreversibly chelate enzymic proteins and destroy the normal functions of enzymes, thus causing damage to immune, metabolic and reproductive systems, and in severe cases, directly causing death (Abu-El-Zahab et al. 2019; Ommati et al. 2019). Besides these toxicities negatively affecting human health, disposal of food due to metal contamination results in great losses to national economies (Li et al. 2017; Rajeshkumar et al. 2018; Yang et al. 2018).

The detection of heavy metals in agricultural environments is the first step to ensuring health and safety in relation to the human diet. Rapid and effective detection of different heavy metal ions is the key to control such pollution. Biosensors for heavy metal detection can provide more rapid and effective advantages of high selectivity and sensitivity, low cost and fast analysis. These advantages can overcome the disadvantages of traditional detection methods, such as spectroscopy, mass spectroscopy and atomic absorption spectroscopy (Deng et al. 2019; Jia et al. 2020; Khodadadi et al. 2019). Biosensors are biological molecules that function by converting reaction signals into detectable signals, and these sensitive molecules act as both signal receivers and converters (Wang et al. 2019). Enzymic proteins, microorganisms, cells, tissues, etc. can act as the sensitive molecules (Mehta et al. 2016).

At present, the many studies on biosensors have primarily focused on microbial cell-based biosensors (Xu et al. 2013). Moreover, according to Yoon’s study, the selectivity of whole-cell bioreporters (WCBs) should be modulated, because the selectivity and sensitivity of these biosensors vary depending on the nature of their promoters and regulatory proteins (Yoon et al. 2018). Unfortunately, because most of the WCBs used in past studies have been genetically modified strains of microbes, they are limited to laboratories or specific environments, where many heavy-metal polluted areas may not be as tenable for application of these WCBs. Therefore, new forms of biosensors need to be developed, and protein sensors that are independent of extracellular cells have emerged at the historic moment.

A variety of potential biosensors for the detection of heavy metal ions are metal-binding proteins that are rich in histidine and L-cysteine amino acids, which help form the protein chelates with metal ions, and have strong affinity for some heavy metals, such as Cd^2+^, Co^2+^ and Cu^2+^ (Tan and Schirmer 2017). Furthermore, with the discovery of the green fluorescent protein in 1962 (Prasher DC^1^,^1992^), fluorescence application in biosensor technology has attracted more attention in current research. In 2019, Lee et al. (2019) constructed a new biosensor by inserting metal-binding loops (MBLs) into a loop region of the enhanced green fluorescent protein (eGFP) to render the eGFP inactive. When exposed to Cd^2+^ and Hg^2+^, the conformation of their recombinant protein changed and consequently, restored the original activity of the eGFP. The concentrations of Cd^2+^ and Hg^2+^ in artificially metal-amended soil and water samples were successfully quantified in the low range of 0–5 μΜ by this novel biosensor, and the accuracy of detection achieved more than 80% for each metal. An *Escherichia coli* sensor harboring eGFP-MBLs could also detect Cd^2+^ and Hg^2+^ in the range of 0–5 μΜ (Kim et al. 2019). Nearly at the same time, another fluorescence-backed biosensor was reported by Soleja et al. 2019. A periplasmic mercury-binding protein, MerP, was designed to be expressed while located between two mutants of the green fluorescent protein to obtain a recombinant protein MerFS for detecting the concentration of Hg^2+^ with high sensitivity and selectivity. Additionally, they constructed a mutant of MerFS, MerFS-51, that lowered the detection limit of Hg^2+^ from 0.210 µM to 1.196 µM. They also reported that MerFS-51 could also detect the intracellular Hg^2+^ concentrations of *Escherichia coli*, yeast and human embryonic kidney (HEK) − 293 T cells.

In this study, we developed a new biosensor consisting of a metal-binding protein and a fluorescence protein to detect Cd^2+^ and other common heavy metals found in agricultural soils and crops. The rice-derived cadmium-binding protein D6A3, which was first identified by our laboratory (Yu et al. 2018), was directly fused with the red fluorescent protein mApple to construct a new biosensor named mApple-D6A3. To determine its efficacy as a biosensor, we first confirmed that mApple-D6A3 fluorescence intensity was linearly correlated with the concentrations of Cu^2+^, Ni^2+^ and Cd^2+^ and observed how variable pH affected intensity. The accuracy of our biosensor in detection of heavy metal ions in artificially amended water or LB medium was more than 80%. Our results demonstrate how a protein biosensor constructed with a fluorescent protein can be effectively applied to detect heavy metal ions in liquid samples.

## Materials and methods

### Materials

Taq DNA Polymerase and Phanta Max Super-Fidelity DNA Polymerase for PCRs were purchased from Vazyme (Nanjing, China). T4 Ligase and restriction endonucleases were bought from TaKaRa (Tokyo, Japan). Plasmid miniprep and DNA gel extraction kits were provided by Axygen (Union City, CA, USA). A nickel-packed column was obtained from General Electric Company (Boston, MA, USA). The bicinchoninic acid (BCA) protein assay kit was obtained from ComWin Biotech (Beijing, China), and the *E. coli* BL21 (DE3) and TOP10 competent cells were from the same company. Analytical reagents were purchased from Sinopharm Chemical Reagent Co. (Beijing, China). Luria–Bertani medium was composed of (L^−1^) 10.0 g NaCl, 5.0 g yeast extract, and 10.0 g peptone, with the pH adjusted to 7.0 with NaOH (5 M); solid LB medium was supplied with 1.5% agar. All strains in this study were cultured at 37° C and agitated at 200 rpm.

### Construction of engineered plasmids and strains

Four plasmids were constructed and their sequencing information are shown in Additional file 1: Table S1. The function and origin of these metal-binding proteins is described in the results section. Overlap PCR (Horton et al. 1989) was used to amplify the complete fragments of mApple-CadR (with primers mApple-F and CadR-R [*Hin*dIII]), mApple-D6A3 (mApple-F and D6A3-R), mApple-CapB (mApple-F and CapB-R) and mApple-MT (mApple-F and MT-R [*Hin*dIII)]. Additional file 1: Table S2 contains the primer information for the PCRs and overlap PCRs. Between fluorescent protein and metal-binding protein coding sequences were two glycine-linked peptides with the codons GGG GGA. After obtaining the correct-sized fragments, mApple-CadR, mApple-D6A3, mApple-CapB and mApple-MT were ligated into the plasmid pET30a (+) at the sites of *Eco*RI (5') and *Hin*dIII (3') to obtain recombinant plasmids pET30a-mApple-CadR, pET30a-mApple-D6A3, pET30a-mApple-CapB and pET30a-mApple-MT (Additional file 1: Table S3). Each recombinant plasmid was transformed into *E. coli* BL21 (DE3) competent cells according to a standard heat shock method (Mandel and Higa 1970). We used the method of inducing expression and purification of the recombinant protein described by (Yan et al. 2018).

### Detection of stability of fluorescence intensity of mApple-D6A3 at different pH values

The partially purified protein mApple-D6A3 was diluted with deionized water to 0.5 mg/mL. The buffers with different pH values were disodium hydrogen phosphate-citric acid buffer (pH 2.2–8.0) and sodium carbonate-sodium hydrogen phosphate buffer (pH 9.16–10.57). We successively added 100 μL each of the protein solution and then different pH buffers into a 96-well microplate, and left it at room temperature for 5 and 20 min. A SpectraMAX M2 microplate reader (Molecular Devices, USA) was used to detect excitation light at a wavelength of 568 nm and emission light at a wavelength of 592 nm. The software used for data processing and analysis was Origin 8.0 (OriginLab Corporation, Northampton, MA, USA).

### Detection of metal ions by mApple and mApple-D6A3

The divalent metal ions CuCl_2_, NiCl_2_, CdCl_2_, CaCl_2_, MgCl_2_, MnCl_2_ and BaCl_2_ were separately dissolved with deionized water to mother liquor concentrations of 10 mM. The proteins, mApple-D6A3 and mApple, were separately diluted to 0.5 mg/mL with 20 mM Tris–HCl (pH 7.4). One hundred microliters of each metal ion solution and deionized water without metal ions (control) were transferred into three wells of a microplate for each solution. Then 100 μL of the two protein solutions were separately added into their own set of each of the metal ion solutions and control in the plate. The plate was left at room temperature for 15 min to measure the fluorescence intensity of the mixed solutions.

### Evaluation of binding ability of mApple-D6A3 to metal ions

The binding ability of mApple-D6A3 to metal ions was evaluated by isothermal titration calorimetry (ITC). The measurements were conducted on a Micro 200 (General Electric Company, USA) with a 300 μL sample cell at 25 ℃. The protein mApple-D6A3 was dialyzed to 40 μM against deionized water and introduced into the sample cell. For binding assays, cells containing the protein or the control (deionized water) were separately titrated with 700 μM of each ion Cu^2+^, Ni^2+^ and Cd^2+^ dissolved in deionized water. No precipitate was produced during the titration. Data were analyzed with the Microcal Origin software (OriginLab Corporation, Northampton, MA, USA).

### Measurement of mApple-D6A3 fluorescence intensity in relation to heavy metal ion concentrations

The partially purified mApple-D6A3 was diluted to 0.5 mg/mL with 20 mM Tris–HCl (pH 7.4) buffer. Stock solutions at 10 mM of each metal, CuCl_2_, NiCl_2_, and CdCl_2_, were used to make a series of diluted solutions at concentrations of 0, 20, 40, 60, 80, 100 and 120 μM for each metal. Each diluted solution of metal ions (100 μL) was added into each microtiter well containing 100 μL of the protein solution. After incubation at room temperature for 15 min, the fluorescence intensity was measured as indicated above.

### Determination of heavy metal (Cu^2+^, Ni^2+^, and Cd^2+^) content in artificially contaminated water and culture medium using mApple-D6A3

The partially purified protein mApple-D6A3 was diluted to 0.5 mg/mL with tap water, and a concentration gradient (20, 40 and 60 μM) of heavy metal solutions was prepared also using tap water as a solvent. The detection method was performed as indicated above.

We also artificially contaminated LB medium with heavy metal ions at a concentration gradient of 0.2 and 0.6 mM. Simultaneously, the engineered strain BL21 (DE3) harboring pET30a-mApple-D6A3 was inoculated into LB medium and cultured at 37 °C while being agitated at 200 rpm. When the OD_600_ of the culture measured between 0.6–0.8, the expression of mApple-D6A3 was induced by adding 0.5 mM IPTG and then further cultured at 16 °C while shaking at 200 rpm for 16–18 h. To obtain the *E. coli* BL21 (DE3) cell bodies, 500 μL of the culture broth was taken and centrifuged at 4 °C, 10,000 rpm for 5 min, and the supernatant solution was discarded. One milliliter of metal ion solution of each of the different concentrations were added to each cell sample and incubated at 37 °C and agitated at 200 rpm for 20 min. Then 200 μL of each mixture was transferred to a microplate to measure fluorescence intensity as indicated above.

## Results

### Expression of screened recombinant sensory proteins

**Fig. 1:**
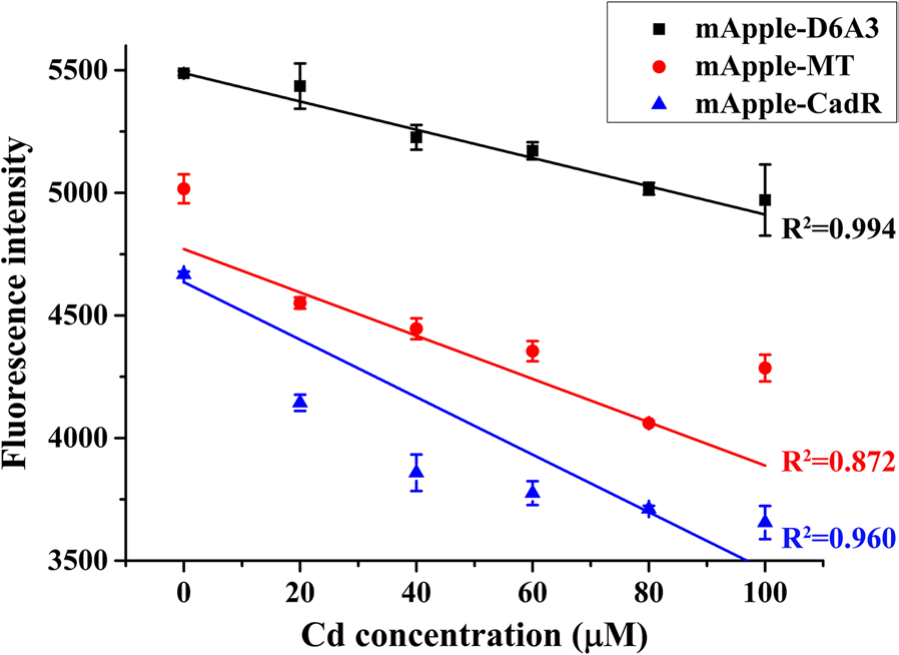
The linear relationships between different sensor proteins and the concentration of Cd^2+^. black box: mApple-D6A3, red circle: mApple-MT, blue triangle: mApple-CadR

Metal-binding proteins, as potentially essential elements of biosensors, have different qualities, stabilities, and abilities to bind to metal ions. In order to determine the best protein as a biosensor that is also suitable for complex and variable external environments, four different metal-binding proteins were screened: CapB, a protein associated cadmium resistance in *E. coli* (Qin et al. 2017); D6A3, a cadmium-binding protein originating from rice (Yu et al. 2018); CadR, a protein derived from the *Pseudomonas putida* S16 and involved in intracellular metal efflux (Lee et al. 2001); and metallothionein (MT), a metal-binding protein derived from mice (Valls et al. 2000). Then the expression of each recombinant protein in *E. coli* BL21 (DE3) was tested. The results comparing our four metal-binding proteins showed that the mApple-CapB protein formed insoluble inclusion bodies, while the soluble proteins of mApple-CadR, mApple-MT, and mApple-D6A3 were successfully expressed (Additional file 1: Fig. S1). When 100 μM Cd^2+^ was added to the recombinant protein solution (0.5 mg/mL), the fluorescence intensity of all three soluble recombinant proteins variably declined, suggesting differential affinity and chelation properties of these recombinant proteins for Cd^2+^. We suspect that the decline may be due to the amount and location of Cd^2+^ binding to the sensor proteins (Khan et al. 2020). Subsequently, the correlations between the increase in the concentration of Cd^2+^ and the decrease in the fluorescence intensity of the three soluble recombinant proteins were examined and produced linear relationships between the two elements. The mApple-D6A3 construct had the highest linear correlation coefficient (R^2^ = 0.994), indicating the strongest relationship with the concentration of Cd^2+^. Coefficients of the other two proteins mApple-CadR and mApple-MT were 0.960 and 0.872, respectively (Fig. 1). Hence, we selected mApple-D6A3 as the best candidate for a metal-sensing protein in this study.

### Detection for multiple metal ions

**Fig. 2:**
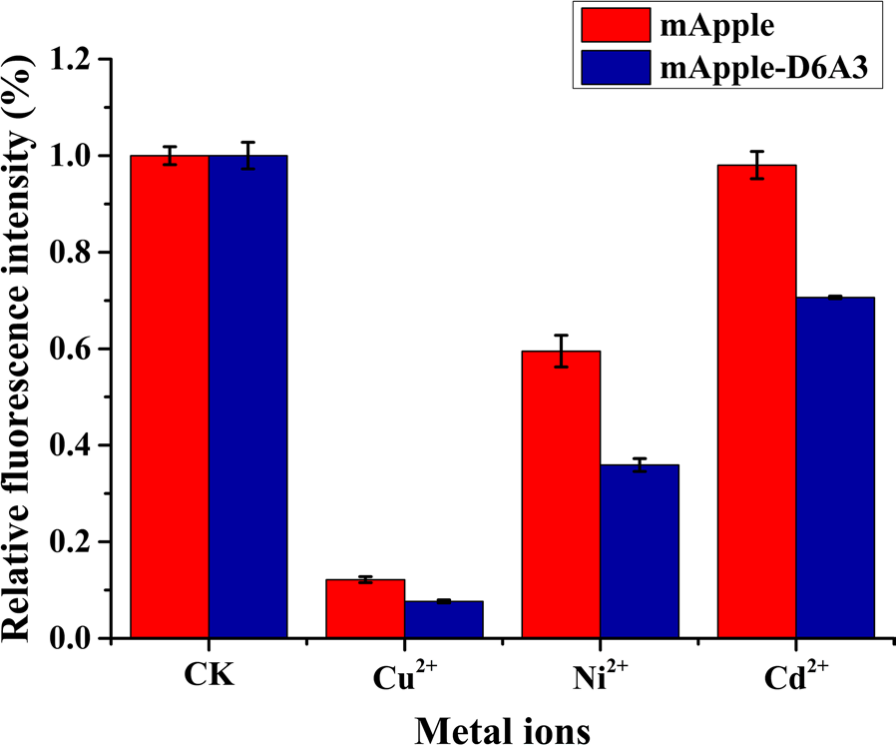
The response of mApple and mApple-D6A3 to Cu^2+^, Ni^2+^ and Cd^2+^

In order to broaden the application range of this biosensor protein mApple-D6A3, the protein was incubated with each of six different divalent metal ions (Ca^2+^, Mg^2+^, Mn^2+^, Ba^2+^, Ni^2+^ and Cu^2+^). Intensity of fluorescence was used to measure the level of response of the sensor protein in detecting each metal ion. The results showed that the fluorescence intensity of mApple-D6A3 did not differ much when Ca^2+^, Mg^2+^, Mn^2+^, or Ba^2+^ was added (Additional file 1: Fig. S2). In contrast, the addition of Cu^2+^, Ni^2+^ or Cd^2+^ reduced fluorescence intensity to 7%, 35% and 70% of the intensity of the control group, respectively (Fig. 2). We then examined the effects of Cu^2+^, Ni^2+^ and Cd^2+^ on the fluorescence intensity of cells transformed with mApple alone. The results show that compared to the control group, the presence of Ni^2+^ reduced the fluorescence intensity of mApple by about 40%, Cu^2+^ almost quenched the fluorescence to about 12%, and Cd^2+^ did not change significantly the fluorescence intensity of mApple (Fig. 2). These results further demonstrate the ability of the recombinant protein mApple-D6A3 to detect heavy metals.

### Effect of pH on fluorescence intensity of mApple-D6A3

**Fig. 3:**
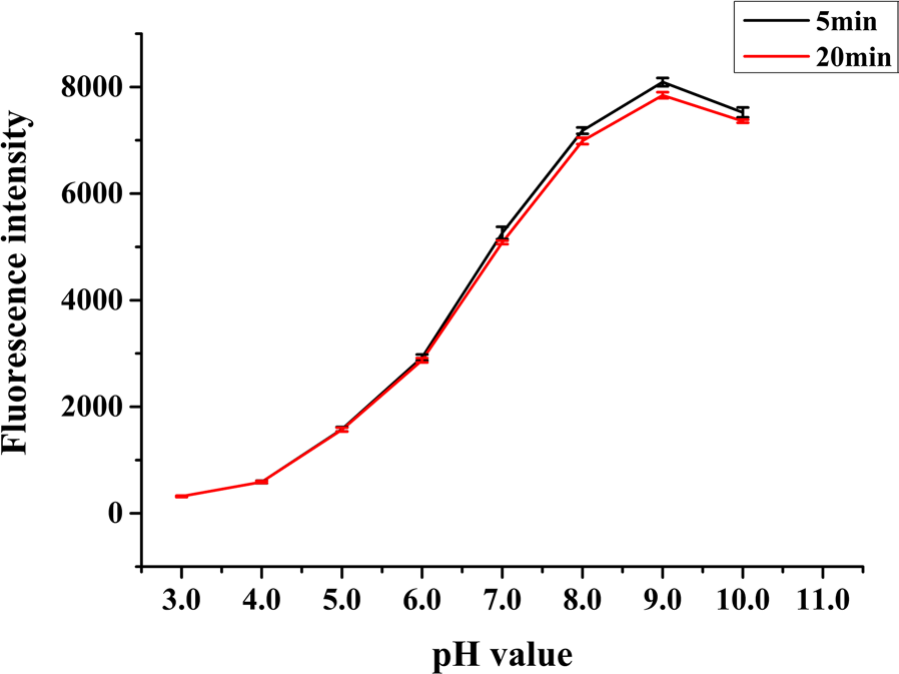
Fluorescence intensity of the mApple-D6A3 protein across a range of pH levels

To determine the stability of the sensor protein mApple-D6A3 acting in potentially complex and changing external environments, we assessed its activity under different pH levels for two different incubation periods. Similarities in fluorescence intensity between the two incubation treatments across the range of pH levels suggest that incubation time had little effect on fluorescence intensity of mApple-D6A3, while pH had significant effect on intensity. Fluorescence intensity of mApple-D6A3 increased with increasing pH between 3.0 and 9.0, decreased slightly between 9.0 and 10.0 and peaking at 9.0 (Fig. 3). The slight decrease in intensity may be caused by partial protein denaturation under alkaline conditions. The results suggested that the fluorescence intensity of the sensor protein was severely hindered under acidic conditions and relatively stable under alkaline conditions. Therefore, the pH of reaction buffers used in further experiments in this study was 9.0.

### Mechanism of interactions between mApple-D6A3 and metal ions

**Fig. 4:**
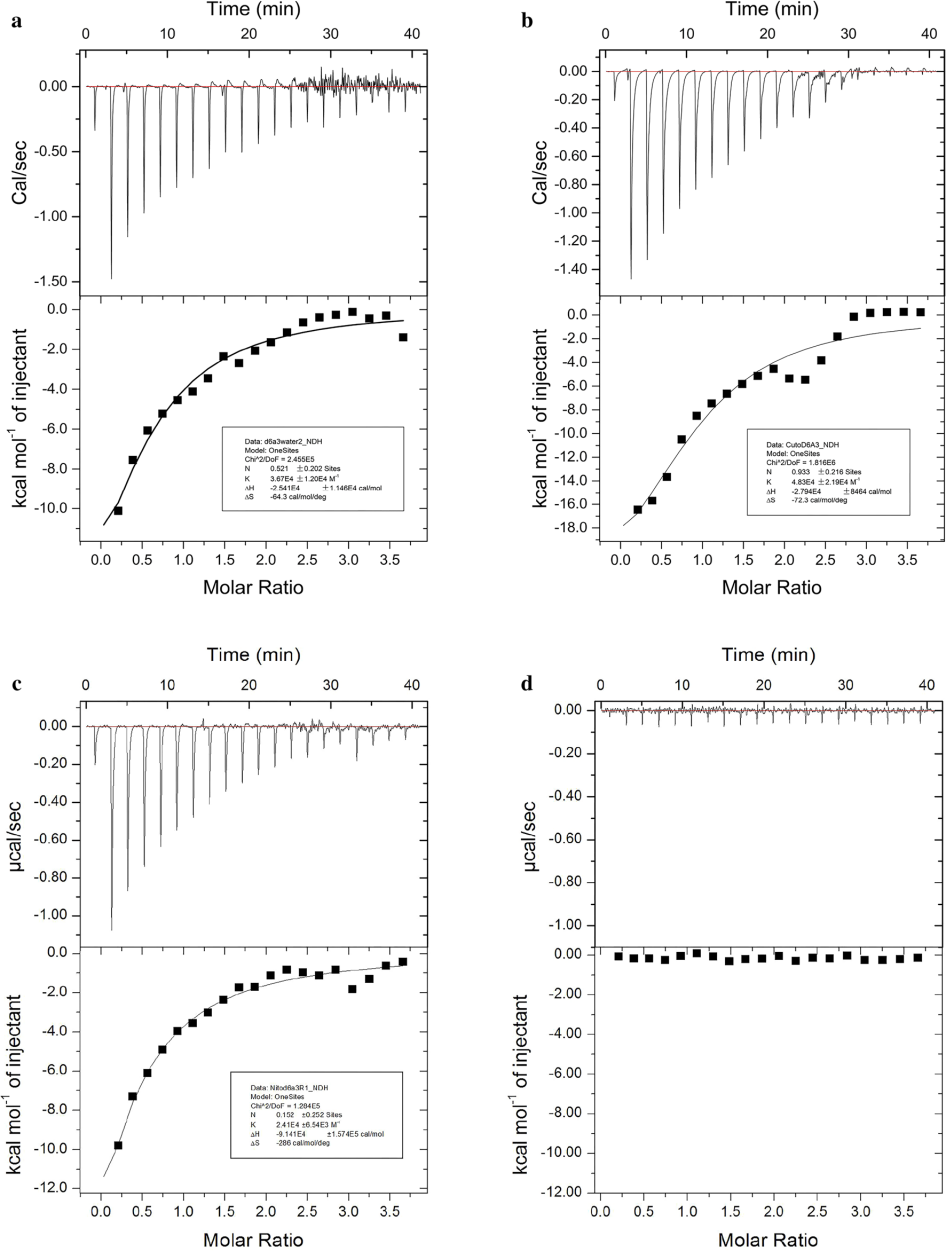
Determination of binding capacity of mApple-D6A3 to heavy metal ions by isothermal titration calorimetry to the metal ions were **a** Cd^2+^, **b** Cu^2+^, and **c** Ni^2+^. **d** Deionized water was the control

Isothermal titration calorimetry experiments were performed to determine the binding properties of sensory proteins to different metal ions (Cd^2+^, Cu^2+^ and Ni^2+^). The results show that, compared with the control group, when a solution of either Cd^2+^, Cu^2+^ or Ni^2+^ was dripped into the sensor protein solution, an obvious exothermic reaction occurred. With the increase of number of drops of the ion solution, the exothermic heat gradually decreased until saturation. The K_D_ values for Cd^2+^, Cu^2+^ and Ni^2+^ were 27.25 ± 8.91, 20.70 ± 9.39 and 41.49 ± 11.26 μM, respectively. The *ΔH* values were − 25410 ± 11460, − 27940 ± 8464 and − 91410 ± 157400 cal/mol, respectively (Fig. 4). The results indicate that the strength of binding capacity of mApple-D6A3 to metal ions ranked as follows: Cu^2+^  > Cd^2+^  > Ni^2+^.

### Biosensor detection of heavy metal ions in soil and sewage

**Fig. 5:**
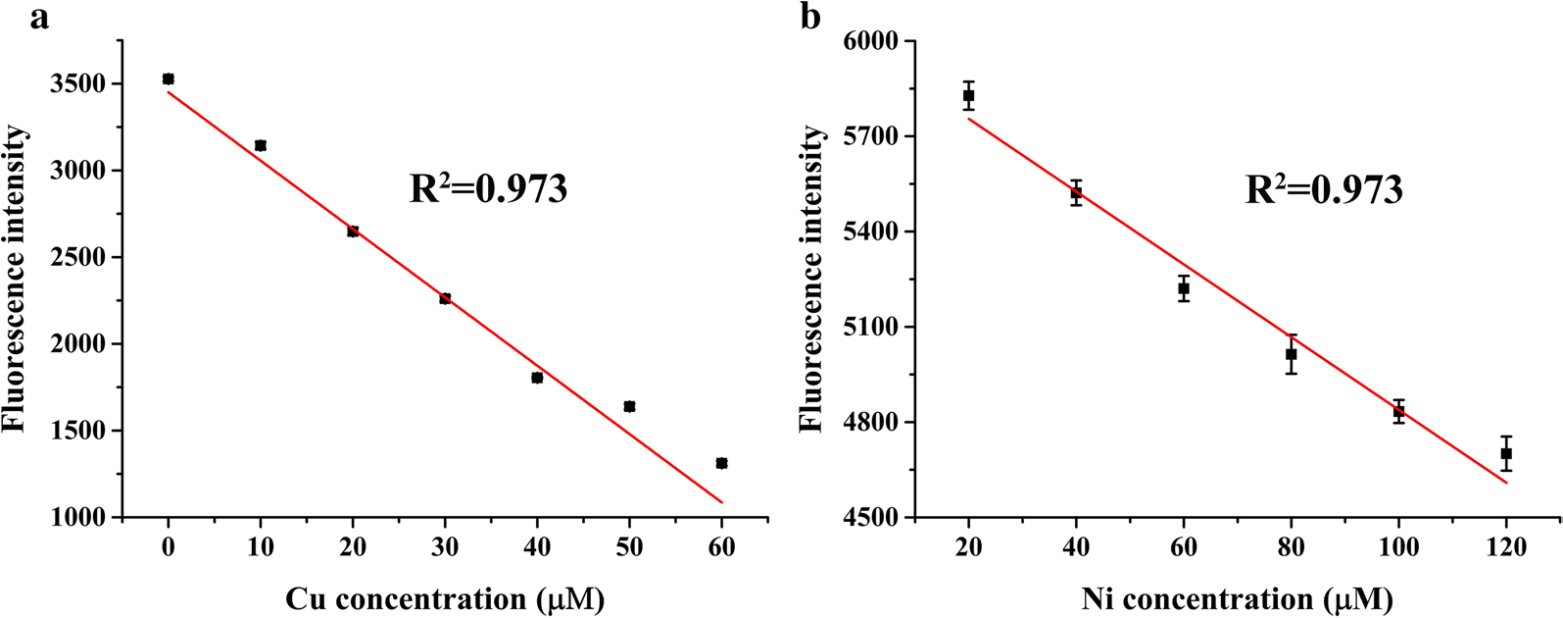
Relationships between mApple-D6A3 and heavy metal ions. The linear relationships between mApple-D6A3 fluorescence intensity and the concentrations of **a** Cu^2+^ and **b** Ni^2+^

As shown in Fig. 1, there was a strong linear relationship between the fluorescence intensity of mApple-D6A3 and concentration of Cd^2+^ (R^2^ = 0.994). There were also highly correlated linear relationships between the fluorescence intensity of mApple-D6A3 and the concentrations of Ni^2+^ and Cu^2+^, where for both metals R^2^ = 0.973 (Fig. 5). Because of these high correlations, we used each of the three heavy metal ions to artificially pollute tap water and culture medium and tested mApple-D6A3′s ability to detect these ions in each substance.

The results show that the concentrations of Cu^2+^, Ni^2+^, and Cd^2+^ in tap water detected by the mApple-D6A3 protein were 18.67 ± 3.74, 21.38 ± 3.11, and 19.27 ± 0.52 μM, respectively. Compared with the standard concentration of 20 μM, the accuracy rates were 93%, 94% and 96%, respectively. The detection accuracy of the 40 μM Cu^2+^- and 60 μM Ni^2+^-tap water solutions also reached more than 80% (Table 1).

**Table 1:** Cu^2+^, Ni^2+^, and Cd^2+^ in tap water detected by the mApple-D6A3 protein

Cu^2+^			Ni^2+^			Cd^2+^		
S	D	A	S	D	A	S	D	A
20	18.67 ± 3.74	93	20	21.38 ± 3.11	94	20	19.27 ± 0.52	96
40	40.69 ± 3.51	98	40	42.55 ± 5.56	94	40	44.56 ± 1.68	90
60	55.1 ± 4.18	92	60	55.53 ± 3.75	93	60	48.85 ± 5.79	81

S: Standard concentration (μM); D: Detection concentration (μM); A: Accuracy (%)

In LB culture samples, the artificially established concentrations of Cu^2+^ (0.2 mM) and Ni^2+^ (0.6 mM) were measured using BL21 cell bodies that expressed the mApple-D6A3 protein. The detected concentrations were 0.23 ± 0.03 mM from the 0.2 mM Cu^2+^ treatment, 0.21 ± 0.04 mM from the 0.2 mM Ni^2+^ treatment, 0.74 ± 0.03 mM from the 0.6 mM Cu^2+^ treatment and 0.60 ± 0.01 mM from the 0.6 mM Ni^2+^ treatment. The accuracy rates were as high as 87%, 93%, 81% and 99%, respectively, indicating that the fluorescent cells have the ability to recognize Cu^2+^ and Ni^2+^ in the LB medium.

The concentrations of Cd^2+^ in the culture medium of treatment concentrations of 0.4 and 0.6 mM were also measured by the fluorescent expression in cells. The former treatment measured at 0.47 ± 0.03 mM and the latter treatment measured at 0.66 ± 0.11 mM; both rates of detection were above 80% (Table 2). Overall, this experiment demonstrated that the sensor protein mApple-D6A3, or its host cell, has the ability to detect heavy metal ions in tap water and LB medium with complex components, with greater than 80% accuracy.

**Table 2:** Cu^2+^, Ni^2+^, and Cd^2+^ in culture medium detected by the mApple-D6A3 host

Cu^2+^			Ni^2+^			Cd^2+^		
S	D	A	S	D	A	S	D	A
0.2	0.23 ± 0.03	87	0.2	0.21 ± 0.04	93	0.4	0.47 ± 0.03	93
0.6	0.74 ± 0.03	81	0.6	0.61 ± 0.01	99	0.6	0.66 ± 0.11	90

S: Standard concentration (mM); D: Detection concentration (mM); A: Accuracy (%)

## Discussion

The metal-binding protein D6A3 had previously been shown to be able to bind Cd^2+^ (Yu et al. 2018). Here, we demonstrated that the biosensor mApple-D6A3 has the ability to bind Cu^2+^ and Ni^2+^ as well. Furthermore, the strongest to weakest binding capacities of mApple-D6A3 to the three divalent metal ions was Cu^2+^  > Cd^2+^  > Ni^2+^, while the strongest to weakest response sensitivity of mApple-D6A3 to the same ions was Cu^2+^  > Ni^2+^  > Cd^2+^. Together, the data indicate that there was no direct association between the response sensitivity and binding capacity of the sensor protein. Perhaps the exposure to metal ions may have altered the conformation of the recombinant protein, thus affecting the position of the fluorescent luminescent group. The position of the chromophore is closely related to changes in fluorescence intensity of the sensor protein (Koker et al. 2018; Krishnamoorthy 2018; Luo et al. 2016).

The biosensor we constructed, mApple-D6A3, is naturally a recombinant protein. In previous work, we attempted to construct a sensor protein using the green fluorescent protein. Unfortunately, the fluorescence of the GFP was quenched by Hg^2+^ readily and thus inadequate for our purposes. The presence of the fluorescent protein mApple not only endowed this recombinant protein with fluorescent properties, it also functioned in enhancing protein stability. In addition, the metal-chelating characteristics of the metal-binding protein D6A3 contributed to the successful construction and application of the biosensor.

The sensor protein mApple-D6A3 detected metal ions in the concentration range of 0–1 mM, which is relatively high (Kim et al. 2019; Lee et al. 2019). However, the limit of reliable detection mainly depends on the sensor’s response to heavy metal ions and noise in the read out system (Leuermann et al. 2019). Nevertheless, we can continue to investigate further the mechanisms of action of mApple-D6A3 on metal ions and combine these future studies with bioinformatics strategies to design mutants with enhanced sensitivity and efficiency for binding heavy metal ions, while minimizing the potential effects that the external environment may have on them, thereby expanding their detection limits.

Every detection method has its benefits and drawbacks. Compared to WCBs, protein biosensors are disadvantaged because of their poor stability and resistance to stress when applied in complex environments. But as a non-living macromolecule, they have the most important advantage of being eco-friendlier. Although our proposed strategy requires further improvements, we creatively combined a red fluorescent protein with a metal-binding protein to promote the development of biosensing technology.

## Supplementary Information

**Additional file 1: Table S1.** sequence information.** Table S2.** Primers used in this study.** Table S3.** Strains and plasmids used in this study.** Fig. S1.** SDS-PAGE of purified mApple-D6A3, mApple-CadR and mApple-MT. M: protein marker. 1: mApple-MT; 2: mApple-D6A3; 3: mApple-CadR.** Fig. S2.** The response of the mApple-D6A3 to other different metal ions.
