# Supplementary material for: Construction of a mApple-D6A3-mediated biosensor for detection of heavy metal ions
Source: AMB Express. 2020 Dec 7;10:213. doi: 10.1186/s13568-020-01154-9 (PMC7721944; doi:10.1186/s13568-020-01154-9)
Supplement: Supplementary file 1 — Additional file 1: Table S1. sequence information. Table S2. Primers used in this study. Table S3. Strains and plasmids used in this study. Fig. S1. SDS-PAGE of purified mApple-D6A3, mApple-CadR and mApple-MT. M: protein marker. 1: mApple-MT; 2: mApple-D6A3; 3: mApple-CadR. Fig. S2. The response of the mApple-D6A3 to other different metal ions. [file 13568_2020_1154_MOESM1_ESM.pdf]

Journal name: AMB Express

Manuscript Title: Construction of a mApple-D6A3-mediated biosensor for detection of heavy metal ions

Author: Yangyang Ji[a, b,1], Feifei Guan[b,1], Xin Zhou[a, b], Xiaoqing Liu[b], Ningfeng Wu[b], Daling Liu[a,2], Jian Tian[b, 2]

a School of life science and technology, Jinan University, Guangzhou，Guangdong 10559, China.

b Biotechnology Research Institute, Chinese Academy of Agricultural Sciences, Beijing 100081, China.

[1] These authors contributed equally to this work

[2] Corresponding authors

*Corresponding author:

Jian Tian; E-mail: tianjian@caas.cn; Tel.: (86) 82106354; Fax: 86-10-82109844.

Daling Liu; E-mail: tldl@jnu.edu.cn.

Additional Tables

Table S1 sequence information

Protein	GenBank	Species	Primers used for amplying the coding sequences	Source PCR template
mApple	HQ423140.1	synthetic construct	mApple-F, mApple-CadR/D6A3/ CapB/MT–R	pET30a-mApple stored in lab
CadR	AEJ15515.1	*Pseudomonas putida* S16	CadR-mApple-F, CadR-R (*Hin*dIII)	pET30a-CadR stored in lab
D6A3	EAZ26583.1	*Oryza sativa Japonica*	D6A3-mApple-F, D6A3-R	pET30a-A4AGZ 4 stored in lab
CapB	CP043211.1	*Escherichia coli* O16:H48	CapB-mApple-F, CapB-R	pUC19-CapB-G FP stored in lab
MT	XP_005079186.1	*Mesocricetus auratus*	MT-mApple-F, MT-R (*Hin*dIII)	pET30a-MT stored in lab

Table S2 Primers used in this study

Primers	Nucleotide Sequence (5'to3')
mApple-F	CGGAATTCATGGTTAGCAAAGGCGAAGAAAACAACATGGC
mApple-CadR-R	CAGTTCGCCAATTTTCATTCCCCCTTTGTACAGTTCGTCCAT
mApple-D6A3-R	GCCACCCTTCTTCCCTTCCCCTCCTTTGTACAGTTCGTCCAT
mApple-CapB-R	AATGGTGGGCGTTTTCATCCCTCCTTTGTACAGTTCGTCCAT
mApple-MT-R	ACTGCAGTTCGGATCCATTCCCCCTTTGTACAGTTCGTCCAT
CadR-mApple-F	ATGGACGAACTGTACAAAGGGGGAATGAAAATTGGCGAACTG
CadR-R(*Hin*dIII)	CCCAAGCTTGTGACCATGGGAACGGC
D6A3-mApple-F	ATGGACGAACTGTACAAAGGAGGGGAAGGGAAGAAGGGTGGC
D6A3-R	CCCAAGCTTGTCATCGTCGTCAGG
CapB-mapple-F	ATGGACGAACTGTACAAAGGAGGGATGAAAACGCCCACCATT
CapB-R	CCCAAGCTTCGCAGCTCTGCTGTCACT
MT-mApple-F	ATGGACGAACTGTACAAAGGGGGAATGGATCCGAACTGCAGT
MT-R(*Hin*dIII）	CCCAAGCTTAGCACAGCAGGTACATTTGTCGG
T7	TAATACGACTCACTATAGGG
T7ter	GCTAGTTATTGCTCAGCGG

Table S3. Strains and plasmids used in this study

Strains or plasmids	Phenotypes or sequences	Source
E.coli TOP10	Cloned strain, Construction of recombinant expression vector pET30a (+)	Stored in lab
E.coli BL21 (DE3)	Expression strain, Expression of recombinant protein (mApple-D6A3,mApple-CadR,mApple-CapB,mApple-MT),	Stored in lab
pET30a（+）	Kanar，*E. coli* TOP10/BL21 (DE3) subtilis shuttle vector, 5422 bp	Stored in lab
pET30a-mApple-CadR	pET30a derivative harbouring mApple-cadR fusion gene, 6564 bp.	This study
pET30a-mApple-D6A3	pET30a derivative harbouring mApple-D6A3 fusion gene, 6825 bp.	This study
pET30a-mApple-CapB	pET30a derivative harbouring mApple-CapB fusion gene, 7722 bp.	This study
pET30a-mApple-MT	pET30a derivative harbouring mApple-MT fusion gene, 6306 bp.	This study

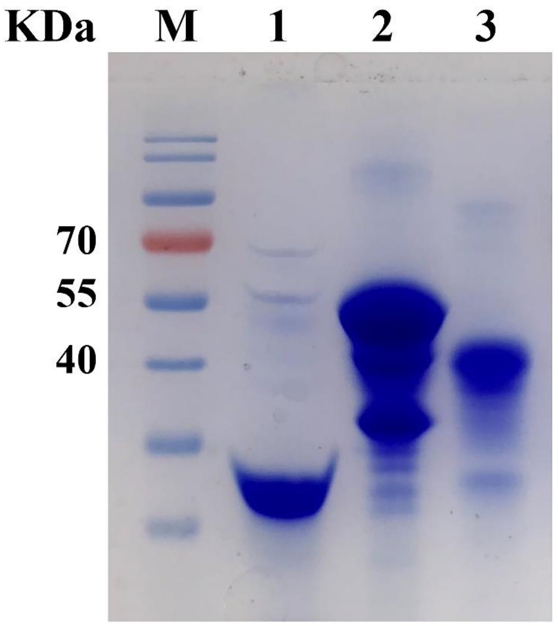

Fig. S1. SDS-PAGE of purified mApple-D6A3, mApple-CadR and mApple-MT.

M: protein marker. 1: mApple-MT; 2: mApple-D6A3; 3: mApple-CadR.

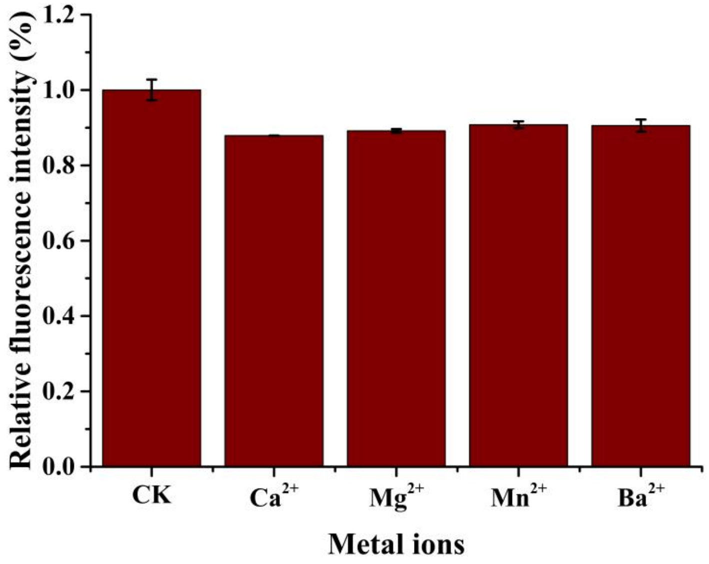

Fig. S2. The response of the mApple-D6A3 to other different metal ions
